# Predicting clinical outcomes using cancer progression associated signatures

**DOI:** 10.18632/oncotarget.27934

**Published:** 2021-04-13

**Authors:** Jared Mamrot, Nathan E. Hall, Robyn A. Lindley

**Affiliations:** ^1^GMDx Group Ltd, Melbourne, Victoria, Australia; ^2^Department of Obstetrics and Gynaecology, Monash University, Clayton, VIC, Australia; ^3^Department of Clinical Pathology, The Victorian Comprehensive Cancer Centre, Faculty of Medicine, Dentistry & Health Sciences, University of Melbourne, VIC, Australia

**Keywords:** innate immunity, biomarker, mutagenesis, oncogenesis, cancer progression

## Abstract

Somatic mutation signatures are an informative facet of cancer aetiology, however they are rarely useful for predicting patient outcome. The aim of this study is to evaluate the utility of a panel of 142 mutation-signature–associated metrics (P142) for predicting cancer progression in patients from a ‘TCGA PanCancer Atlas’ cohort. The P142 metrics are comprised of AID/APOBEC and ADAR deaminase associated SNVs analyzed for codon context, strand bias, and transitions/transversions. TCGA tumor-normal mutation data was obtained for 10,437 patients, representing 31 of the most prevalent forms of cancer. Stratified random sampling was used to split patients into training, tuning and validation cohorts for each cancer type. Cancer specific machine learning (XGBoost) models were built using the output from the P142 panel to predict patient Progression Free Survival (PFS) status as either “High PFS” or “Low PFS”. Predictive performance of each model was evaluated using the validation cohort. Models accurately predicted PFS status for several cancer types, including adrenocortical carcinoma, glioma, mesothelioma, and sarcoma. In conclusion, the P142 panel of metrics successfully predicted cancer progression status in patients with some, but not all cancer types analyzed. These results pave the way for future studies on cancer progression associated signatures.

## INTRODUCTION

Cancer is a leading cause of human mortality worldwide and the incidence of cancer is expected to rise as our average life expectancy increases [1–3]. Yet, despite many recent advances in treatment, the immense socio-economic burden of cancer persists [1–3]. A key strategy for reducing the burden of cancer is to personalize treatment regimes to optimize patient outcomes [4]. Currently less than 25% of patients benefit from personalized care [5, 6] and efforts to increase adoption and utility are ongoing, for example by incorporating novel biomarkers into existing treatment methods. Effective personalized cancer treatment requires a detailed understanding of the aetiology, physiology and molecular biology of the cancerous cells. However, many of the mechanisms driving cancer progression are still not fully understood, such as the causes, effects, and patterns of DNA mutation in oncogenesis.

As cancer develops, many mechanisms and endogenous cellular processes cause mutations in DNA. A predominant endogenous DNA mutagen is the orthologous family of proteins known as deaminases, specifically the activation-induced cytidine deaminase/apolipoprotein B editing complex (AID/APOBEC) family of enzymes [7–11] and putatively adenosine deaminases acting on RNA (ADARs) [12–14]. These enzymes mutate DNA and/or can edit RNA [15–17] by binding to specific target motifs. For example, the binding motif for APOBEC3G is two consecutive cytosines (“CC”) and deamination of the second cytosine results in another nucleotide being incorporated e.g., “CT” [9]. Deaminase binding domains are typically highly specific: the deaminase enzyme AID also deaminates cytosines (“C”) predominantly, however it binds at WRC loci (W = A/T, R = A/G) [18, 19].

Analyzing somatic mutations coinciding with known deaminase binding motifs can signal aberrant activity of that specific deaminase and compromised DNA repair in cells [8, 9, 20, 21]. Furthermore, the accumulation of specific deaminase-associated mutations in a patient can provide valuable information on how the cancer has developed [20–23], and in specific cases provide information on the rate of progression of the disease and likely response to specific treatments [24–28]. Despite clinical utility in a handful of examples, quantification of deaminase-associated DNA mutations currently does not provide actionable information for the majority of cancer types [29, 30]. However, further classification and analysis of deaminase-associated DNA mutations can reveal additional information. This has been previously shown using metrics such as strand bias [31]; codon context (the position of the mutated nucleotide within the codon, relative to the transcript start site and read 5’ to 3’ on the non-transcribed strand) [13]; and the number and ratio of transitions and transversions in deaminase-associated variants [32]. For example, higher strand bias and transition/transversion ratios have been observed in inherited disease-associated genetic variants [32].

**Figure 1:**
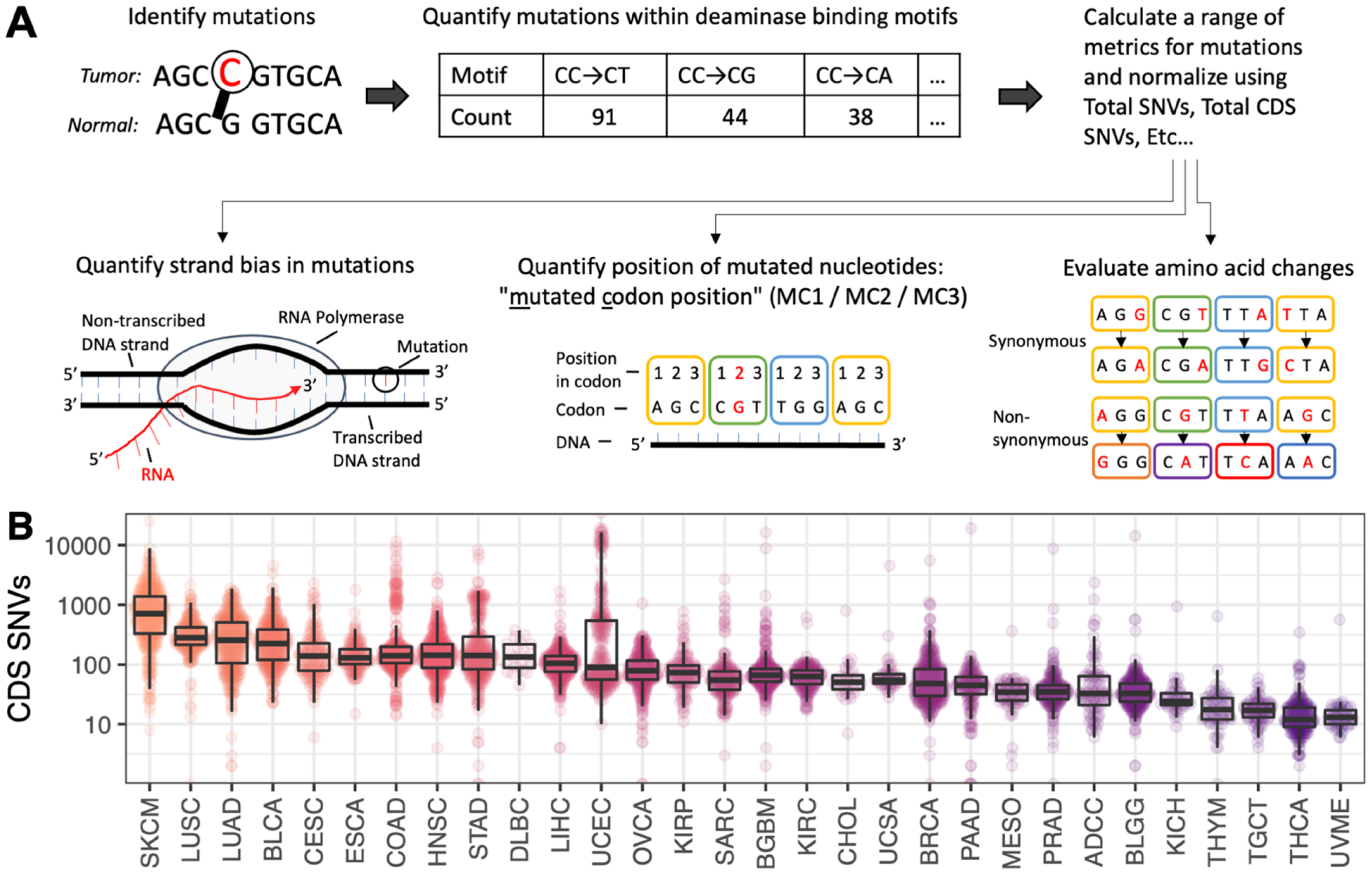
(**A**) Schematic of the key concepts involved in generating output from the P142 metric panel. Each metric in the P142 panel is described in full in Supplementary Table 1. These metrics are derived from tumor-normal single nucleotide variants and cover a range of sequence motifs, incorporating strand bias, codon context, transitions/transversions and synonymous/non-synonymous status. (**B**) Distribution of SNVs contained within the coding sequence (CDS) regions of the genome. Cancer types are ordered and colored from highest to lowest mutation burden.

Antecedent research has shown that, when combined with the codon-context of the targeted motifs for both cytosine and adenosine deaminases as described by Lindley [13], deaminase-associated mutation signatures can be used to stratify patients with high-grade serous ovarian cancer into long-term and short-term survivors [14]. In this study, the proposed molecular model of AID/APOBEC and ADAR mutagenesis advances existing models by implicating the open transcription bubble and transcription elongation complex as illustrated by Lindley [12] (Figure 1A). In this model, deamination targets are presented in C-site motifs both in the displaced ssDNA of the non-transcribed strand, and may also be accessed in the exposed ssDNA of the transcribed strand at annealed RNA:DNA hybrids assisted via the action of the RNA exosome [33]. ADAR A-site deamination targets are present in WA-motifs in the dsRNA stem loops of the nascent pre-mRNA, and in the RNA and DNA A-site moieties in the RNA:DNA hybrid, assisted by the reverse transcriptase activities of DNA Polymerase-eta (Pol η) [31, 34, 35]. This presumptive model is based on known deaminase targets and their role in oncogenesis, combined with the mechanisms underlying reverse transcription. This model contributes to the rationale underpinning the development of the panel of cancer progression associated metrics used in this study.

Whilst the utility of deaminase-associated metrics is yet to be fully realized, it is our view that this approach may ultimately be used to improve the predictive accuracy of a range of emerging genomic diagnostics in an ‘additive’ manner. Several other approaches have also been used to stratify cancer patients into long- and short-term survivors [36–39]. Methods such as analyzing gene expression changes and pathway enrichment [40], protein abundance and localization [41], single nucleotide polymorphisms (SNPs) [42, 43], altered RNA splicing [44], metabolites or other analytes [45] have been used to stratify patients. Models combining these approaches with clinical data such as patient age, sex, treatment and histopathology can achieve relatively high predictive accuracy [46]. The P142 panel is fundamentally different to these existing methods and may provide a new source of cancer progression biomarkers. This is the first application of these deaminase-associated metrics for predicting cancer progression and patient outcomes.

The aim of this study is to evaluate the efficacy of a panel of 142 deaminase-associated metrics (herein referred to as the ‘P142’ panel) for predicting the rate of cancer progression in patients selected from The Cancer Genome Atlas (TCGA) PanCancer Atlas cohort. The P142 metrics evaluated in this study are defined and described in Supplementary Table 1. We hypothesize that the P142 markers associated with AID/APOBEC and ADAR deamination and codon reading frame context can be used to predict cancer progression for patients with a range of different cancer types.

## RESULTS

### Application of the P142 metric panel and patient categorization

The P142 panel of metrics was applied to every patient eligible for inclusion in the study (see Figure 1A; *n* = 5,903) and the results were collated into patient profiles. Each patient profile contains 142 values: one for each metric in the panel. Patient profiles were grouped by cancer type and categorized according to their Progression Fee Survival (PFS) status, labelled as either “Low PFS” or “High PFS”. The median PFS for each cohort and the corresponding PFS threshold used to delineate “Low PFS” patients from “High PFS” patients are presented in Table 1, along with the number of patients in each group. For example, the PFS threshold for TCGA patients with adrenocortical carcinoma (ADCC) is 24 months: patients in the “Low PFS” category presented with cancer progression/recurrence before 24 months (*n* = 39), and patients in the “High PFS” category did not progress before 24 months (*n* = 46) (Table 1).

**Table 1:** A summary of the patient cohorts and key parameters included in the study

Cancer type	Cancer type abbreviation	Median PFS (months)	PFS threshold (months)	No. patients included in analysis	No. patients above PFS threshold	No. patients below PFS threshold
Adrenocortical Carcinoma	ADCC	27.60	24	85	46	39
Glioblastoma Multiforme	BGBM	5.92	6	306	161	145
Bladder Urothelial Carcinoma	BLCA	17.98	18	291	157	134
Brain Lower Grade Glioma	BLGG	27.81	24	290	168	122
Breast Invasive Carcinoma	BRCA	46.62	60	321	211	110
Cervical Squamous Cell Carcinoma	CESC	31.27	30	134	79	55
Cholangiocarcinoma	CHOL	7.96	9	32	18	14
Colorectal Adenocarcinoma	COAD	29.06	24	310	202	108
Diffuse Large B-Cell Lymphoma	DLBC	32.01	30	20	14	6
Esophageal Adenocarcinoma	ESCA	10.55	12	141	83	58
Head and Neck Squamous Cell Carcinoma	HNSC	25.97	24	351	190	161
Kidney Chromophobe	KICH	87.24	60	45	37	8
Kidney Renal Clear Cell Carcinoma	KIRC	45.96	48	264	200	64
Kidney Renal Papillary Cell Carcinoma	KIRP	34.29	36	166	127	39
Liver Hepatocellular Carcinoma	LIHC	12.89	12	254	122	132
Lung Adenocarcinoma	LUAD	23.87	24	320	163	157
Lung Squamous Cell Carcinoma	LUSC	31.56	36	283	188	95
Mesothelioma	MESO	12.00	12	64	16	48
Ovarian Serous Cystadenocarcinoma	OVCA	16.04	18	294	137	157
Pancreatic Adenocarcinoma	PAAD	12.33	12	161	130	31
Prostate Adenocarcinoma	PRAD	37.00	36	222	156	66
Sarcoma	SARC	17.80	18	202	128	74
Skin Cutaneous Melanoma	SKCM	28.59	30	371	219	152
Stomach Adenocarcinoma	STAD	16.57	18	264	145	119
Testicular Germ Cell Tumors	TGCT	40.01	42	73	46	27
Thyroid Carcinoma	THCA	39.73	36	217	175	42
Thymoma	THYM	45.63	48	62	44	18
Uterine Corpus Endometrial Carcinoma	UCEC	35.13	36	257	161	96
Uterine Carcinosarcoma	UCSA	11.64	12	51	28	23
Uveal Melanoma	UVME	27.52	24	52	34	18
TOTALS				5903	3585	2318

Details include cancer type and cancer type abbreviation, the median Progression Free Survival (PFS) time, the corresponding PFS threshold, and the total number of patients in each PFS status group (determined to be either “High PFS” or “Low PFS” using the PFS threshold).

### Distribution of variants in coding regions of the genome

Somatic single-nucleotide variants (SNVs) in protein coding regions (CDS) were quantified for each patient. The distribution of CDS SNVs for each cancer type are shown in Figure 1B. The highest average CDS SNV burden was observed in skin cutaneous melanoma and lung cancers (SKCM, LUAD, LUSC). In comparison, the observed SNV burden was more than 10-fold lower in thyroid cancers, testicular germ cell carcinoma and uveal melanoma (THYM, TGCT, THCA and UVME).

### Correlation between somatic variant burden and progression free survival

**Figure 2:**
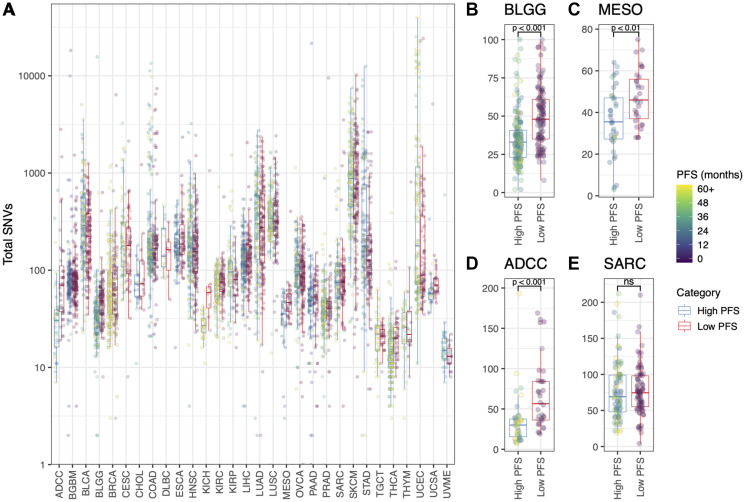
(**A**) Total number of SNVs (log_10_ scale) for each patient, grouped by cancer type and colored by Progression Free Survival (months). “High PFS” = above the PFS thresholds listed for each cancer type in Table 1; “Low PFS” = below the PFS threshold listed in Table 1. Also shown is the total number of SNVs (linear scale) for (**B**) the BLGG cohort, (**C**) the MESO cohort, (**D**) the ADCC cohort, and (**E**) the SARC cohort. *T*-tests were used to statistically evaluate the difference between the “High PFS” and “Low PFS” patient groups (B, C, D, E; ^*^ *p* < 0.05).

A statistically significant correlation between total SNV burden and patient PFS status was not found for the majority of cancer types (see Figure 2). However lower grade glioma (BLGG, Figure 2B), mesothelioma (MESO, Figure 2C) and adrenocortical carcinoma (ADCC, Figure 2D) had significantly more mutations in patients with “Low PFS” vs those with “High PFS” (*p < 0.05*). This trend was not seen in sarcoma (SARC, Figure 2E; *p > 0.05*). Other individual metrics in the P142 panel were similarly weak predictors of patient PFS status in different cancer types (Supplementary Figure 1).

### Cross validation and predictive accuracy of machine learning models

Patients grouped by cancer type were split using stratified random sampling into training, tuning and validation cohorts: 75%, 10% and 15% of patients respectively. These cohorts retained the same approximate ratio of “High PFS” and “Low PFS” patients as the original patient groups. Two cancer type groups (DLBC and KICH) had insufficient patient numbers (i.e., zero “High PFS” or “Low PFS” patients in the tuning or validation cohorts) and were excluded from further analysis.

**Figure 3:**
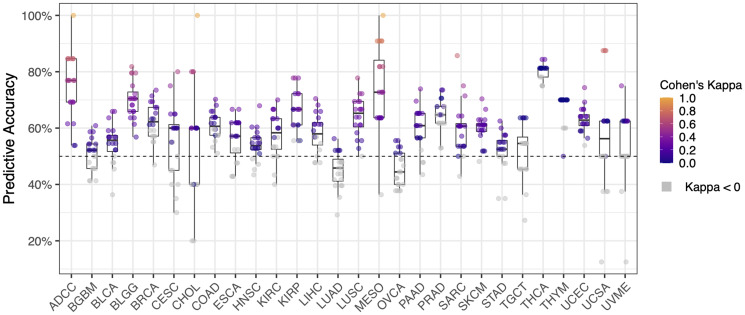
Predictive accuracy of machine learning cross validation (20 rounds) for each cancer type (n = 28). Cohen’s Kappa represents the deviation of the observed accuracy from the expected accuracy for each cohort of patients. Cohen’s Kappa is traditionally evaluated as described in Landis and Koch [74]: values < 0 = not different to random chance, 0–0.20 = slight agreement, 0.21–0.40 = fair agreement, 0.41–0.60 = moderate agreement, 0.61–0.80 = substantial agreement, and 0.81–1 = almost perfect agreement (i.e., perfect predictive accuracy on validation data). Cohen’s Kappa values less than zero are not statistically different to random chance (colored grey).

For the validation cohorts, the average predictive accuracy for machine learning (ML) models across all cancer types was 60% ± 1.2% (mean ± SD; 20 rounds of cross validation per cancer type), higher than expected according to random chance (i.e., 50%). Overall, 59.1% of ML models had a Cohen’s Kappa value > 0 (Figure 3). As shown in Figure 3, the highest predictive accuracy was seen in ML models for adrenocortical carcinoma, mesothelioma and cholangiocarcinoma (ADCC, MESO, CHOL), which predicted the PFS category of validation patients with 100% accuracy. A selection of ML models achieved > 80% predictive accuracy, including models for adrenocortical carcinoma, lower grade glioma, cervical squamous cell carcinoma, cholangiocarcinoma, mesothelioma, sarcoma, thyroid carcinoma, and uterine carcinosarcoma (ADCC, BLGG, CESC, CHOL, MESO, SARC, THCA, UCSA). Overall, one or more ML models achieved a predictive accuracy > 70% in the majority of cancer types in the TCGA (20/28 cancer types analyzed).

### Kaplan–Meier survival curves for each cancer type

**Figure 4:**
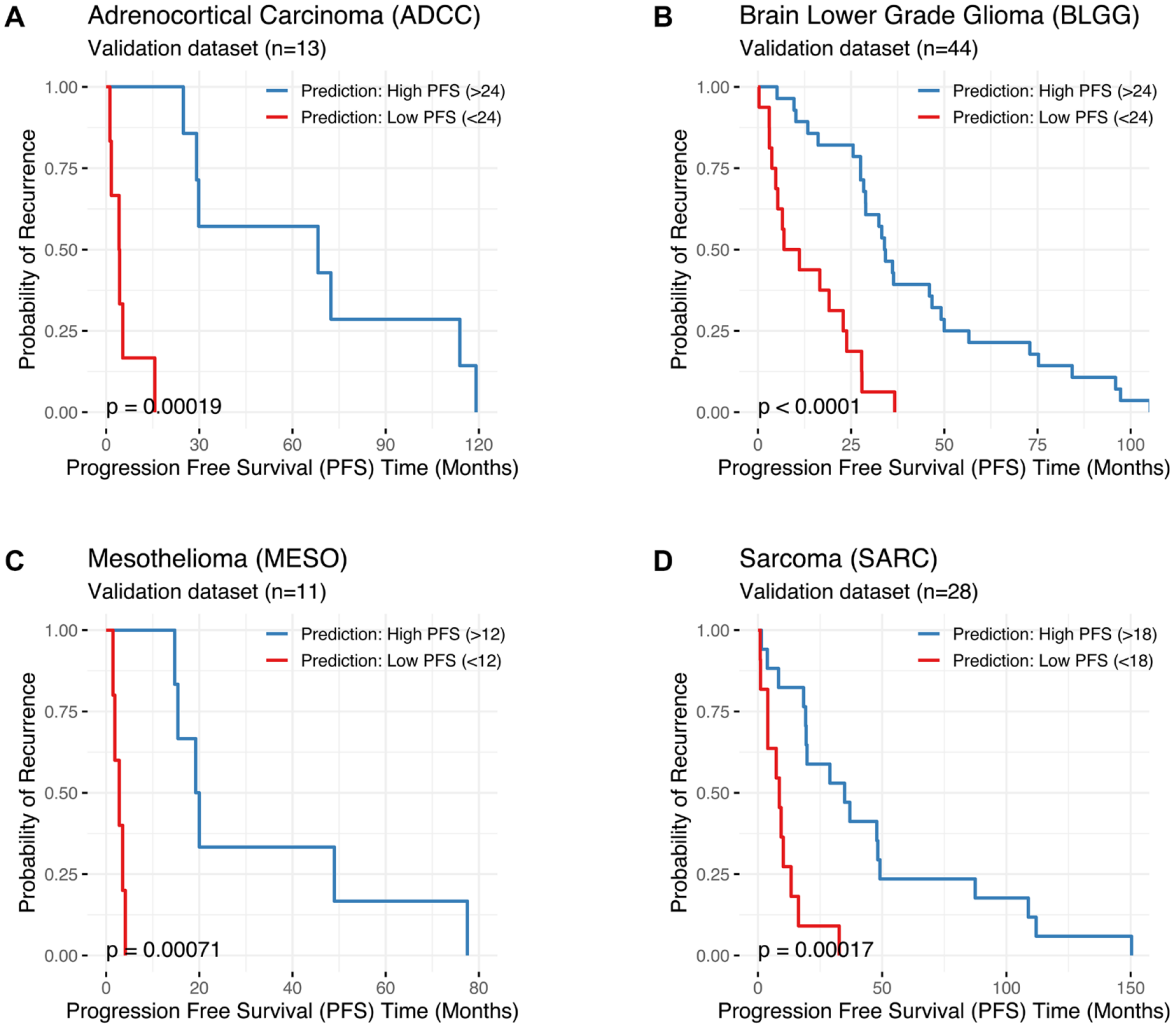
Kaplan–Meier plots comparing the ‘actual’ Progression Free Survival (PFS in months; X axis) of patients predicted to be “Low PFS” (PFS below the threshold, shown in red) versus patients predicted to be “High PFS” (PFS above the threshold, shown in blue) for (**A**) Adrenocortical Carcinoma (ADCC), (**B**) Lower Grade Glioma (BLGG), (**C**) Mesothelioma (MESO), and (**D**) Sarcoma (SARC). Results were generated using the best machine learning (ML) model for each cancer type (i.e., the model with the highest predictive accuracy and Cohen’s Kappa value). Cancer type, the number of patients in the validation cohort, and the PFS threshold used to delineate “Low PFS” from “High PFS” are shown. Statistical comparisons between groups were conducted using log-rank tests (significance: ^*^ *p* < 0.05). Kaplan–Meier plots for all 28 cancer types are presented in Supplementary Figure 2.

A significant difference in the probability of recurrence (the ‘actual’ PFS) between patients predicted as “Low PFS” compared to those predicted as “High PFS” is seen in Kaplan–Meier plots for a selection of cancer types (Figure 4; *p* < 0.05). Kaplan–Meier plots generated by applying the best performing ML model to the respective validation cohorts for each of the 28 cancer types are shown in Supplementary Figure 2. ML models with poor predictive accuracy showed little separation between lines (e.g., lung adenocarcinoma; Supplementary Figure 2N). This is irrespective of the individual patient’s predicted PFS status (“Low PFS” or “High PFS”), as the actual PFS does not correlate with the predictions. Overall, a significant difference in the probability of recurrence (the ‘actual’ PFS) in each validation cohort was observed between patients predicted as “Low PFS” compared to patients predicted as “High PFS” for the majority of cancer types in the TCGA (20/28 cancer types analyzed; *p* < 0.05).

### Evaluation of the chosen PFS thresholds

Additional ML models were created for each cancer type using predetermined PFS thresholds of 6, 12, 24, 36, 48 and 60 months to evaluate the appropriateness of the PFS thresholds used in the study (listed in Table 1). Cross validation was performed (10-fold for each PFS threshold) and Cohen’s Kappa values were used to compare models for each cancer type (Supplementary Figure 3). ML models could not be obtained when the PFS threshold led to insufficient patient numbers in a category (i.e., zero “High PFS” or “Low PFS” patients in the tuning or validation cohorts), for example, Supplementary Figure 3B: ML models were not generated at 48 and 60 months PFS for the BGBM cohort, as only 4 patients with that cancer type survived past 48 months. In the 28 different cancer types investigated, the predetermined PFS threshold (either 6, 12, 24, 36, 48 or 60 months) that was closest to the threshold used in the study (listed in Table 1) had the highest Cohen’s Kappa value and predictive accuracy, confirming the PFS thresholds used in the study were appropriate.

## DISCUSSION

The TCGA PanCancer Atlas has greatly improved our understanding of cancer biology, leveraging comprehensive genomic, epigenetic, and transcriptomic analyses across a broad range of tumor types. Here, we used TCGA whole exome sequencing data to evaluate the utility of genomic metrics associated with deaminase mutagenesis (P142) for predicting the PFS status of patients from 28 cancer types. Machine learning (ML) models generated using the P142 metric panel accurately categorized patients as “High PFS” (above the PFS threshold) or “Low PFS” (below the PFS threshold) with up to 100% predictive accuracy and with Cohen’s Kappa values above 0.4 for 11 cancer types. Nevertheless, the ML models used did not accurately categorize patients in all cancer types. In addition to existing methods of quantifying somatic mutations with putative deaminase binding motifs, incorporating additional physiological characteristics such as strand bias, codon context and transitions/transversions, provides new insights and novel genomic biomarkers that can be used to predict PFS status in cancer patients. These findings support our hypothesis that deaminase-associated metrics can be used for predicting patient outcome and provides a foundation for further development of diagnostic and prognostic tests.

An advantage of the methods developed for this study is that the P142 metrics implicate a broad range of physiological and molecular mechanisms associated with mutagenesis. For example, some deaminases are known to preferentially target the transcribed or non-transcribed strand of a transcript [18, 47–50]. One P142 metric that incorporates this specificity is #15: “cds:A3B_T-C-W G>A motif %” (Supplementary Table 1), which evaluates the proportion of mutations that occur within a known APOBEC3B target motif that we classify as G>A. As this metric denotes a mutation of “G” within an APOBEC3B motif, the mutation occurred at C>T on the opposite strand, which is a measure of strand bias [45]. Another example is the position of the mutated nucleotide in the codon, which evaluates synonymous verses non-synonymous amino acid changes and implicates factors relating to DNA structure and conformation [13]. This mechanism is associated with many of the P142 metrics, for example #7: “cds:A3G_C-C- MC3%” (Supplementary Table 1), which calculates the proportion of mutations that occur within the APOBEC3G motif (“CC”) at the 3rd position of a codon (“MC3”). Leveraging physiological and molecular insights associated with deaminase mutagenesis can reveal differences between patients despite similar overall deaminase signature profiles. This is a key point of difference between this study and alternative approaches such as the “mathematical” deaminase signature quantification methods used by others (e.g., [11, 51–53]).

Despite a clear correlation between the P142 metrics and patient outcome in the majority of cases, predictive accuracy was low for 8 of the 28 cancer types investigated. A potential explanation for this result is that deaminase expression is highly tissue-specific and perhaps these cancer types have lower deaminase activity or expression. This is a potential direction for future investigation. It should be noted that high expression of deaminase enzymes does not necessarily cause more mutations in DNA unless repair pathways are compromised. Recent investigation into the overexpression of APOBEC3B in cells has found a negligible increase in mutations *in vitro*. Yet, in cells that are p53-compromised a significantly higher number of mutations with the expected APOBEC motif were observed when APOBEC3B was overexpressed [54]. Thus, despite expression of deaminases varying greatly between cell/tissue types, the relationship between expression and phenotype is complex and cannot be easily quantified by measuring gene or protein expression or by counting SNVs that occur within deaminase binding motifs. This is another potential explanation for the low level of deaminase-associated mutations seen in some cancer types, such as TGCT and UVME, which do not typically have compromised DNA repair machinery.

An ongoing research question in oncology is the clinical utility of Tumour Mutational Burden (TMB) as a biomarker for patient survival. TMB is increasingly being incorporated into genomic testing (diagnostic and prognostic) and it is the leading biomarker used to predict patient outcome after immune checkpoint inhibitor therapy [55–58]. High TMB is typically associated with positive response to immunotherapy (and subsequently higher PFS), yet in cancer types such as adrenocortical carcinoma and lower grade glioma (ADCC, BLGG) a lower mutation burden was associated with longer PFS (see Figures 3 and 4). This finding was also reported in a recent study by Alghamri et al. [59]. There are several potential explanations for this observation. For instance, TMB may not relate to patient outcome unless the patient is treated with immunotherapy, or perhaps TMB is not a useful biomarker in the cancer types described. Nevertheless, the link between TMB and patient outcome in cancer types such as ADCC and BLGG may be useful for customizing a panel of metrics to increase predictive accuracy and utility.

Machine learning methods were used in this study to circumvent inherent correlations between individual P142 metrics. For example, normalizing metric values using the total number of mutations occurring within a specific motif is a known source of potential bias. The XGBoost decision tree ensemble algorithm was chosen specifically to mitigate this issue. Despite high predictive accuracy in the final model, we did not observe individual metrics to be strong predictors of patient PFS status, and the distribution of patient scores for each metric when compared between cancer types and PFS status was highly variable (Supplementary Figure 1). An explanation for these observations is that each metric made a small contribution to the final overall prediction, and the contribution was dependent on cancer type. For example, the distribution of patient scores for a specific metric in the four highlighted cancer types with the highest predictive accuracy were not always found to be concordant (Supplementary Figure 1C). The XGBoost algorithm is well-suited to this scenario as it combines bagging and boosting algorithms to build weak learner models initially, then improves the learner models as training progresses [60]. Overall, the findings of this study support using the XGBoost algorithm over other analytical methods.

Genomic instability is the accumulation of somatic mutations and chromosomal alterations within cellular lineages [61]. This is a hallmark of cancer, and often these somatic mutations do not have a discernable source as they are caused by mechanisms such as oxy-radicals (e.g. oxidation damage creating G → A mutations via Guanine → 8-Oxoguanine → Adenosine), or exposure to radiation [62]. Mutations without a discernable source are not targeted to specific genomic motifs (unlike deaminase-associated mutations), though there are regions that appear to be more susceptible than others [63, 64]. We have previously speculated that the combinatorial association of different deaminase isoforms, homodimers and heterodimers may moderate deaminase targeting specificity and contribute to this accumulation of mutations as cancer progresses [65]. This phenomenon is referred to in the literature as ‘trained’ innate immunity and experimental evidence is now being generated to examine the potential contribution of deaminase isoforms and dimers to genomic instability [66–68]. Regardless of the underlying mechanism, the accumulation of somatic mutations without a discernable source may reduce our ability to resolve deaminase-mediated mutation signatures in specific regions of the genome. Establishing baseline levels of 8-Oxo-G–associated mutations and other non-deaminase–associated mutations in future studies would address this potential confounding factor.

Notable limitations exist in TCGA genomic sequencing data that may have affected the results of this study. For instance, sparse personal information and clinical history, missing metadata, predominantly Caucasian samples, and a single timepoint for almost every patient may have reduced the overall predictive power and limit translation of the results obtained. Datasets generated from prospective, purpose-designed studies could ameliorate these caveats and may provide a more accurate estimate of the predictive power and potential utility of the P142 panel. Furthermore, prospective studies could eliminate the effects of other confounding factors inherent to NGS technology, for example by obtaining high tumor purities, using high depth sequencing (e.g., 500X), ensuring accurate tumor subtyping, and implementing new technologies and bioinformatics tools. Creating homogenous datasets and augmenting the P142 panel with additional biomarkers may help to improve the predictive accuracy for those cancer types with poorer predictive outcomes as well as provide further insight into tissue-specific deaminase mutagenesis.

In conclusion, we have identified a correlation between cancer patient outcomes and changes in metrics associated with deaminase mutagenesis in some, but not all, cancer types investigated. Potential molecular explanations for this observation are based on our evolving understanding of dysregulated deaminase DNA mutagenesis, disrupted DNA-RNA repair pathways and subsequent aberrant protein production. Further investigation using prospective, purpose-designed studies would likely improve the efficacy of machine learning models and provide a more accurate evaluation of the potential utility of this approach. This study provides a basis for further development of biomarker panels based on metrics associated with deaminase mutagenesis for predicting cancer progression and patient outcome.

## MATERIALS AND METHODS

### Data source

The Cancer Genome Atlas (TCGA) is a collaboration between the National Cancer Institute (NCI) and the National Human Genome Research Institute (NHGRI). A prominent TCGA initiative is the ‘PanCancer Atlas’ project, conducted by the Multi-Center Mutation-Calling in Multiple Cancers (MC3) network, which includes genomic tumor-normal mutation exome data for 10,437 tumors from 33 of the most prevalent types of cancer. The TCGA PanCancer Atlas has been widely used to improve our understanding of cancer biology across a broad range of tumor types. This well-characterized cohort of patients has specific features and characteristics that enable the application of state-of-the-art machine learning methods: primarily the size of the dataset and homogeneity of metadata.

The results shown here are in whole or part based upon data generated by the TCGA Research Network: https://www.cancer.gov/tcga. Data access is via the NIH Genomic Data Commons (https://gdc.cancer.gov/access-data/data-access-processes-and-tools). Data visualization can be conducted using the cBioPortal for Cancer Genomics (https://www.cbioportal.org/) [69, 70].

Patients without a valid Progression Free Survival (PFS) and patients without any detected somatic mutations were excluded from analysis. Somatic mutations were obtained for 9,433 patients (30 different cancer types) in VCF format (https://samtools.github.io/hts-specs/VCFv4.1.pdf).

Genomic metrics included in the P142 panel relate to mutational burden, deaminase binding motifs, incidence of tumor/normal single nucleotide mutations, and reading-frame context of the codon triplet (i.e., if a codon contains a mutation, what is the position of the mutated codon (MC): 1, 2 or 3 as read 5’ to 3’). The panel also contains additional metrics, such as those related to transitions/transversions and strand bias. The metrics included in the P142 biomarker panel used in this study are defined in Supplementary Table 1.

### Data analysis

**Figure 5:**
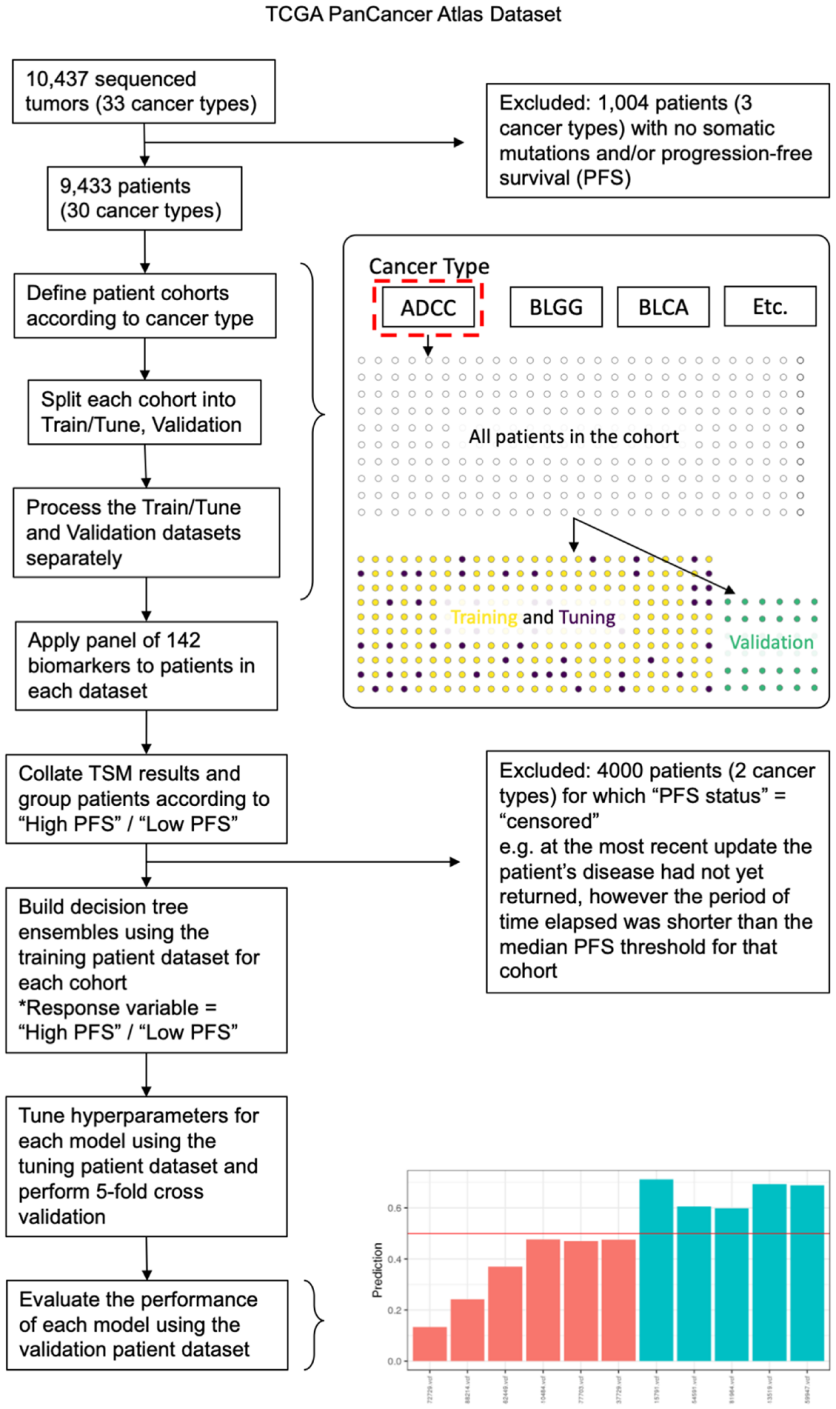
Data analysis pipeline outlining key data processing, machine learning analysis, and validation steps used in the study. Exclusion criteria are described further in the Methods. Patients were grouped according to cancer type and were split into training, tuning and validation cohorts using a stratified semi-random approach. The P142 panel was then applied to patients in each cohort separately and the TSM results (the collective output of the panel) were collated. For each cancer type, the XGBoost algorithm was used to train and tune models using the training and tuning cohorts, then the final model was evaluated using the validation cohort. This process was repeated (20 rounds) using a different patient split in each case. The barplot illustrates predictions for validation patients (prediction values between 0 and 1) with patients predicted to be “Low PFS” below the red line and patients predicted as “High PFS” above the red line. Bars are colored according to their actual PFS status: red = “Low PFS” and green = “High PFS”.

SNVs for each patient were processed using the ‘Targeted Somatic Mutation’ (TSM) platform as described in Lindley et al. [14]. Schematics of the data processing pipeline are provided in Figure 1A and Figure 5. For each patient, the bases surrounding each somatic SNV were identified, and mutations with defined motifs were quantified to create a patient profile. Patient profiles were then collated, and patients were grouped by cancer type for analysis. Patients were annotated according to their Progression Free Survival (PFS) time in months. PFS is defined as the period of time from the date of diagnosis until the date of the first occurrence of a new tumor event (NTE), locoregional recurrence, distant metastasis, a new primary tumor, or death with tumor. Patients were categorized as “High PFS” or “Low PFS” according to the median PFS (months) from the TCGA PanCancer cohort rounded to the nearest 6-month increment. The PFS threshold was adjusted for three cancer types (BRCA, COAD, KICH) to more closely reflect PFS thresholds previously reported in the literature. The thresholds used to delineate “High PFS” from “Low PFS” patients for each cancer type are shown in Table 1.

Machine learning models were built to predict membership to either the “High PFS” or “Low PFS” groups, and a nested cross validation (CV) was performed to evaluate predictive performance. For each cancer type, data was split into partitions using the createDataPartition function from the caret package (https://topepo.github.io/caret/data-splitting.html) [71]. For each CV iteration (*n* = 20), one partition was used to train the model (using 75% of patients), one partition was used for tuning hyperparameters (random search, https://mlr.mlr-org.com/reference/makeTuneControlRandom.html [72]) (using 10% of patients), and one partition was used to evaluate predictive accuracy for validation (using 15% of the total number of patients that were removed from the patient pool before data processing, training or tuning was conducted).

Patients were randomly allocated to each partition for each iteration (random seed), and a “gbtree” booster was applied with a “binary:logistic” objective function [60]. Hyperparameter values, or range of values, included an “eta” between 0.1 and 0.3, “gamma” = 0, “subsample” = 0.8, “max depth” between 4 and 12, “minimum child weight” between 2 and 8, “colsample by tree” between 0.5 and 1, and up to 250 “rounds” of training with “early stopping” after 50 rounds with no improvement in error rate. The accuracy of each trained classifier for predicting the class labels (“High PFS” or “Low PFS”) of patients in the validation partition was then evaluated for each iteration. Evaluation included calculation of sensitivity, specificity, positive predictive value, negative predictive value, balanced accuracy and Cohen’s Kappa values.

In addition to the clinically relevant PFS thresholds used to delineate between “High PFS” and “Low PFS” patients, further training/tuning/validation was conducted at 6, 12, 24, 36, 48, 60 months PFS for each cancer type (Supplementary Figure 3).

### Data visualization and statistics

Data analysis and visualization was conducted using R (v4.0.2) and python (v3.7.7). The R package Janitor v1.2.1 (https://garthtarr.github.io/meatR/janitor.html) was used to correct variable names. Data was partitioned using caret v6.0-85 (https://topepo.github.io/caret/) [71], and models were trained using XGBoost v0.90.0.2 (https://xgboost.readthedocs.io/) [60]. Hyperparameters were tuned using MLR v2.17.0 (https://github.com/mlr-org/mlr) [72] and data was visualized using functions from the tidyverse v1.3.0 (https://github.com/tidyverse/tidyverse) [73] and XgboostExplainer v0.1 (https://github.com/AppliedDataSciencePartners/xgboostExplainer). Cohen’s Kappa statistic was defined as *κ* = $\frac{po-pe}{1-pe}\;\;=\;\;1-\frac{1-po}{1-pe}$ where *po* is the observed predicted accuracy, and *pe* is the expected predictive accuracy, per Landis and Koch [74]. The magnitude of Cohen’s Kappa was evaluated as: values < 0 = not different to random chance, 0–0.20 = slight agreement, 0.21–0.40 = fair agreement, 0.41–0.60 = moderate agreement, 0.61–0.80 = substantial agreement, and 0.81–1 = almost perfect agreement (i.e., perfect predictive accuracy on validation data). Survival curves were estimated using the Kaplan–Meier method and compared with a log-rank test using the Survival R package v3.2-3 (https://github.com/therneau/survival) [75].

### Novelty and significance

This is the first study to predict cancer progression in TCGA patients with 28 cancer types using a panel of metrics associated with AID/APOBEC and ADAR deaminase mutagenic processes.

## SUPPLEMENTARY MATERIALS




