# Supplementary material for: Predicting clinical outcomes using cancer progression associated signatures
Source: Oncotarget. 2021 Apr 13;12(8):845–58. doi: 10.18632/oncotarget.27934 (PMC8057277; doi:10.18632/oncotarget.27934)
Supplement: Supplementary file 1 [file oncotarget-12-845-s001.pdf]

Predicting clinical outcomes using cancer progression associated signatures

SUPPLEMENTARY MATERIALS

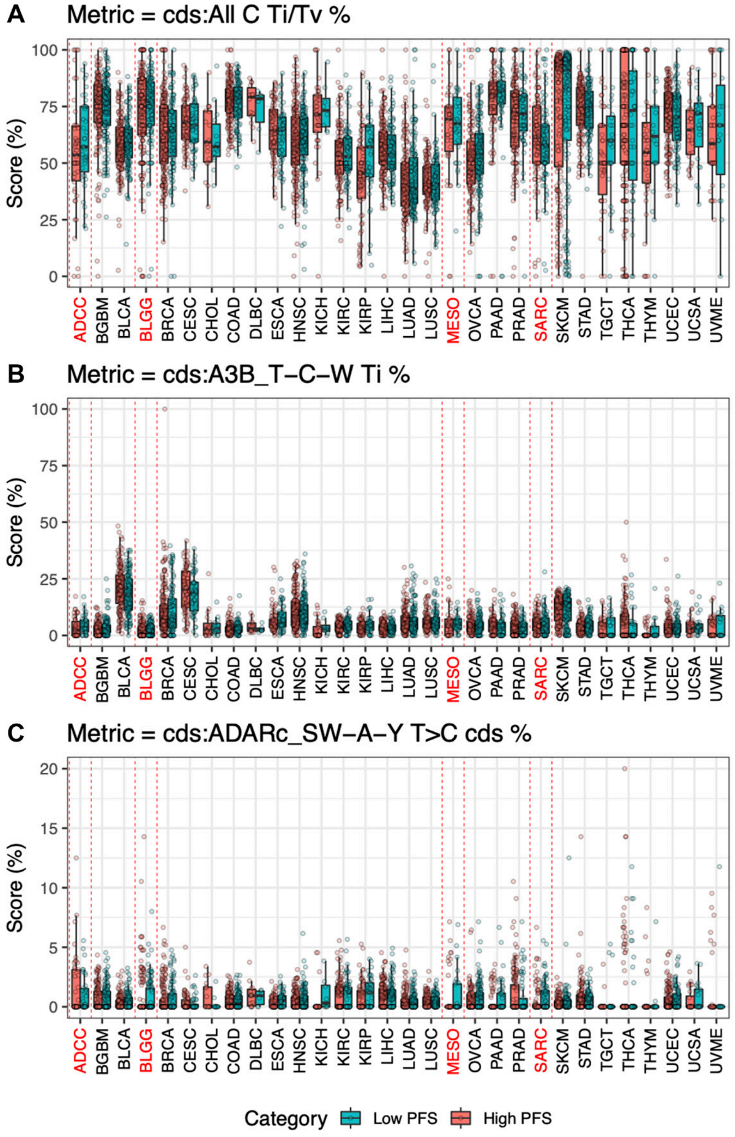

Supplementary Figure 1: Patient values ("Scores") for three representative metrics. Metrics are defined and described in Supplementary Table 1. Patients are grouped and colored by PFS status, defined as above/below the PFS threshold for each cancer type per Table 1. Cancer types with the highest predictive accuracy are highlighted in red (ADCC, BLGG, MESO, SARC).

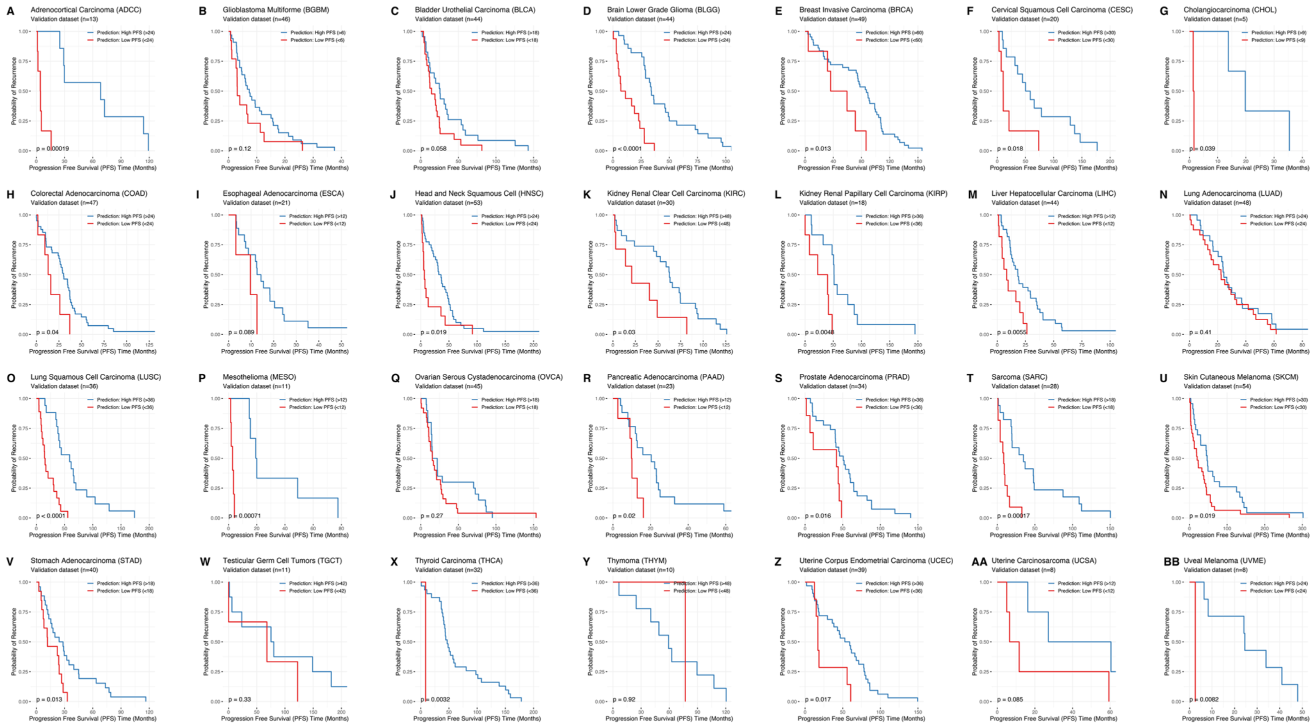

Supplementary Figure 2: Kaplan–Meier plots comparing Progression Free Survival (PFS) of patients predicted to be “Low PFS” (PFS below the threshold, shown in red) versus patients predicted to be “High PFS” (PFS above the threshold, shown in blue) for all cancer types. The type of cancer and acronym are displayed (heading), along with the number of patients in the validation cohort. The PFS threshold used to delineate “Low PFS” patients from “High PFS” for each of the selected models. *P*-values were calculated using log-rank tests (significance: $^{*}p < 0.05$).

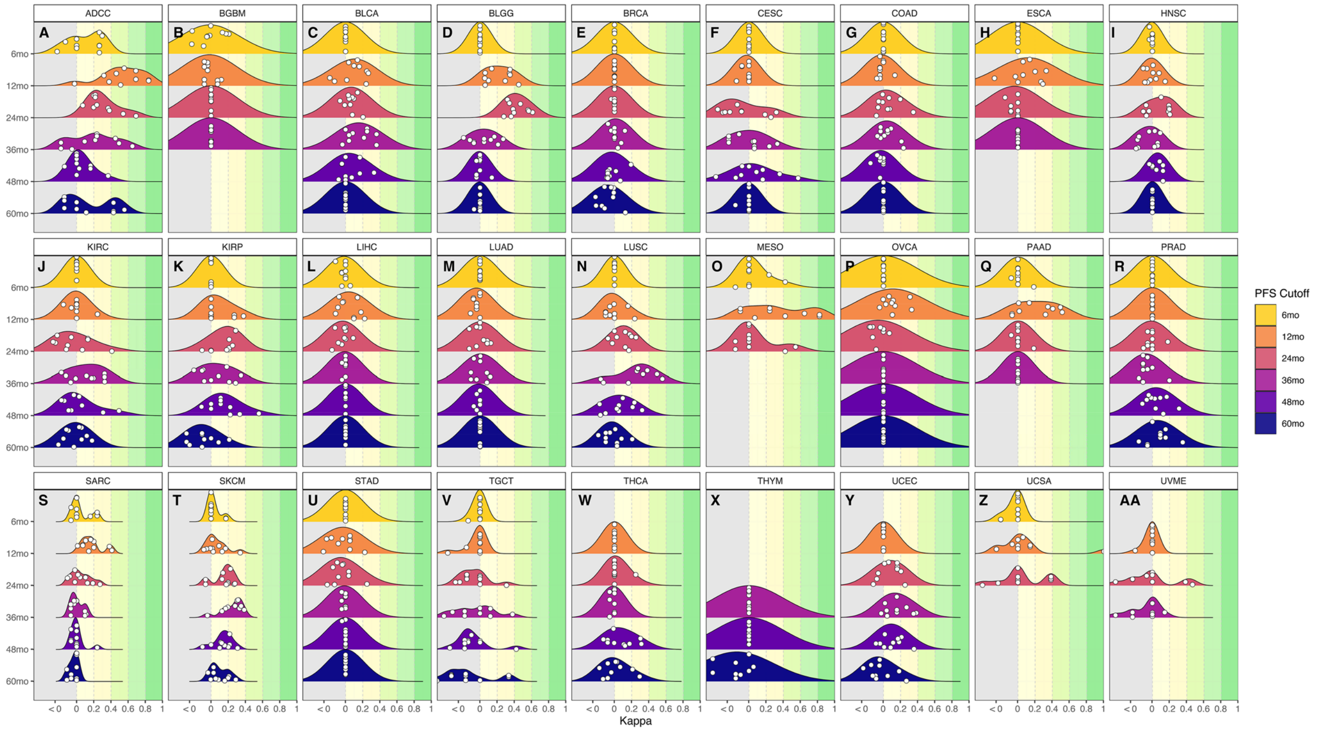

Supplementary Figure 3: Distribution of Cohen's Kappa values for each cancer type (*n* = 28) for specified PFS thresholds: 6, 12, 24, 36, 48, 60 months (10-fold cross validation for each cancer type). Curves that are not shown have zero patients above or below the threshold in either the tuning or validation group. For example, all MESO patients have PFS < 36 months and Cohen's Kappa cannot be calculated for PFS thresholds > 36 months. Cohen's Kappa represents the deviation of the observed accuracy from the expected accuracy for each cohort of patients. Cohen's Kappa is traditionally evaluated as described in Landis and Koch [74]: values < 0 = not different to random chance, 0–0.20 = slight agreement, 0.21–0.40 = fair agreement, 0.41 - 0.60 = moderate agreement, 0.61–0.80 = substantial agreement, and 0.81–1 = almost perfect agreement (i.e., perfect predictive accuracy on validation data). Cohen's Kappa values less than zero are not statistically different to random chance (colored grey). The data show that: ADCC performs best with at PFS cutoff of 12 months (median threshold = 24), BLGG at 24 months (median threshold = 24), SARC at 12 months (median threshold = 18), and SKCM at 36 months (median threshold = 30).

Supplementary Table 1: Details of all 142 CPAS panel metrics describing the name, region, motif and how the metric is calculated. See Supplementary Table 1
